# Differences in accuracy of height, weight, and body mass index between self-reported and measured using the 2018 Korea Community Health Survey data

**DOI:** 10.4178/epih.e2022024

**Published:** 2022-02-19

**Authors:** Yoonsil Ko, Sunhye Choi, Jisoo Won, Yeon-Kyeng Lee, Dong-Hyun Kim, Seon Kui Lee

**Affiliations:** 1 Division of Chronic Disease Control, Bureau of Chronic Disease Prevention and Control, Korea Disease Control and Prevention Agency, Cheongju, Korea; 2 Division of Healthcare Association Infection Control, Bureau of Healthcare Safety and Immunization, Korea Disease Control and Prevention Agency, Cheongju, Korea; 3 Department of Social and Preventive Medicine, Hallym University College of Medicine, Graduate School of Public Health, Chuncheon, Korea

**Keywords:** Community Health Survey, Obesity, Measures, Self-reported

## Abstract

**OBJECTIVES:**

This study aimed to determine an effective survey method for the accurate calculation of obesity prevalence by comparing the self-reported and measured height, weight, and body mass index (BMI) using the 2018 Korea Community Healthy Survey (CHS) data.

**METHODS:**

Raw data from the 2018 CHS were used to analyze the differences, correlation, and agreement between self-reported and measured height, weight, and BMI.

**RESULTS:**

The self-reported height was over-reported than the measured height (0.59 cm greater for men and 0.71 cm greater for women), while the self-reported weight was under-reported than the measured weight (0.55 kg less for men and 0.67 kg less for women). Subsequently, the self-reported BMI was under-estimated (0.35 kg/m^2^ lower for men and 0.49 kg/m^2^ lower for women) compared with the measured BMI. The kappa statistic and agreement between measured and self-reported values per BMI category (underweight, normal, overweight, and obesity) were 0.82 and 79.6%, respectively.

**CONCLUSIONS:**

The prevalence of obesity should be calculated using the measured values provided in the CHS in order to promote local health projects based on accurate evidence.

## INTRODUCTION

Overweight and obesity are defined as abnormal or excessive accumulation of fat that may impair one’s health [1]. In general, obesity is indirectly measured based on the body mass index (BMI, kg/m^2^), which is calculated as weight in kilograms divided by height in meters squared. BMI is used to classify overweight (≥ 25 kg/m^2^) and obesity (≥ 30 kg/m^2^) in adults.

As of 2016, more than 1.9 billion adults worldwide aged ≥ 18 years were overweight, and more than 650 million of them, accounting for 13% of the world’s adult population, were obese (men: 11%, women: 15%). This number has increased almost three-fold compared with that reported in 1975 [1]. In Korea, as of 2019, the prevalence of obesity among adults (BMI ≥ 25 kg/m^2^) was 33.8% (≥ 19 years old, standardized), showing a continuous increase [2]. Obesity is a key risk factor for the development of non-communicable diseases such as type 2 diabetes, cardiovascular disease, hypertension, stroke, and various cancers, and the comorbidities of obesity not only increases the socioeconomic burden, but also decreases the quality of life [3]. The World Health Organization announced the “Global strategy on diet, physical activity, and health” during the 57th Annual Meeting in 2004. This agency is making continuous efforts to prevent and manage obesity, a global health problem [4]. As obesity is a preventable disease [1], continuous policies should be established to investigate the exact prevalence of obesity, and effective preventive strategies should be implemented.

In Korea, the National Health and Nutrition Examination Survey (NHANES) and Community Health Survey (CHS) were conducted to assess the regional and national prevalence of obesity. The data obtained from these surveys were used as evidence for the establishment and evaluation of the national and local government policies to prevent and manage obesity. In the NHANES, the prevalence of obesity was calculated by measuring the height and weight of 10,000 individuals. The CHS targeted 230,000 individuals in order to calculate the self-reported obesity rate since 2008 due to convenience and cost. However, in 2018, body measurements were obtained to calculate the prevalence of obesity.

According to several previous studies, BMI measurements based on self-reported data were used instead of the actual measurements in many large-scale epidemiological studies for their relative convenience and efficiency. However, such self-reported data were shown to be inaccurate as they contain errors such as underreported weight and overreported height by the participants [5-10]. In certain studies, the correlation coefficient between self-reported and actual values was greater than 0.8 [10-19]. In studies conducted in Korea examining the accuracy of self-reported height and weight, it was recommended that the actual measurement values should be used for evaluation of obesity [20] and the height, weight, and obesity rate must be calculated based on the actual data [10]. Additionally, other studies reported that the self-reported values were accurate for use in clinical and public health studies; however, the studies were only conducted in visitors and inpatients of hospital examination centers [19,21]. Therefore, this study conducted a comparative analysis of the prevalence of obesity calculated using the self-reported and measured values of height and weight from the data of the 2018 CHS to suggest a more accurate method for calculation of obesity prevalence.

## MATERIALS AND METHODS

This study was conducted using raw data from the 2018 CHS. This survey was conducted in 254 public health centers nationwide. Each public health center visits 900 individuals for interviews every year from August to October in order to investigate the health of local residents. In the 2018 survey, a total of 228,340 individuals participated. Those who “refused to respond” or responded “do not know” and the outliers (height: < 50 or > 200 cm; weight: < 20 or > 130 kg; and BMI: < 10 or > 50 kg/m^2^) were excluded; hence, a total of 214,640 participants were included in the final analysis, of whom 183,211 participants obtained their own body measurements. Individuals who could not stand still and pregnant women were excluded from undergoing body measurements.

The 2018 CHS was the only survey in which self-reported and actual body measurements of height and weight were simultaneously investigated. Prior to the body measurements, the participants were asked the following question: “What are your height and weight?”

To measure the height and weight during household visits, convenient tools without a large range of error for measurements were selected. The ultrasonic extensometers (InLab S50; InBody Co., Seoul, Korea), and CAS HE-58 (CAS, Yangju, Korea) were used to evaluate height and weight, respectively. The height was measured twice with the participant in a standing position, and the mean value was used for analysis. The weight was measured once. The reliability and effectiveness of the ultrasonic extensometer have been previously demonstrated [22].

### Statistical analysis

SAS version 9.4 (SAS Institute Inc., Cary, NC, USA) was used to perform all statistical analyses. In this complex sample study, a stratified analysis was conducted by gender and age groups (19- 29, 30-39, 40-49, 50-59, 60-69, and ≥ 70 years old). A p-value of less than 0.05 was considered significant. Paired t-test was conducted to calculate the mean and 95% confidence interval (CI) and compared the differences between self-reported and measured values of height, weight, and BMI. Pearson’s correlation analysis was conducted to determine the correlation between the self-reported and measured values, and Bland-Altman analysis was conducted to compare the differences between the two values. The participants were categorized into four BMI groups based on the self-reported and measured values ( < 18.5, 18.5-22.9, 23.0-24.9, and ≥ 25.0 kg/m^2^), and the kappa statistic was assessed to evaluate the agreement by a group. To compensate for the possible errors of measured values obtained using the ultrasonic extensometer, 0.4 cm was subtracted from the height for analysis.

### Ethics statement

This study was not subject to deliberation by the research ethics committee because it was conducted directly or commissioned by the state or local government to review and evaluate public welfare or service programs (Enforcement Rule of Bioethics and Safety Act, Article 2).

## RESULTS

### Participants’ general characteristics

The general characteristics of individuals who participated in both the self-administered questionnaire survey and body measurements and those who participated in the self-administered questionnaire survey, but not in the body measurements were compared.

Young men participants who had high income, a high level of education, a professional or office occupation, were unmarried, *dong* area residents, had a second-generation household type, and were obese (based on self-report) were significantly more likely to complete the self-administered questionnaire and not participate in body measurements (Table 1).

### Comparison of self-reported and measured height, weight, and body mass index values

**Figure 1.**
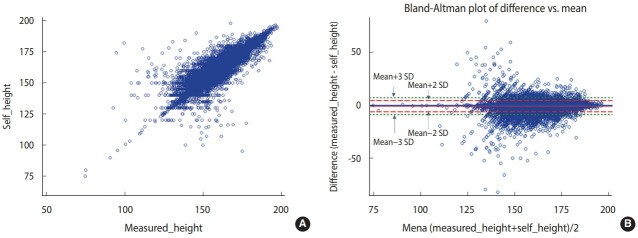
Correlation distribution and Bland-Altman plot of the measured (A) and self-reported (B) height values.

**Figure 2.**
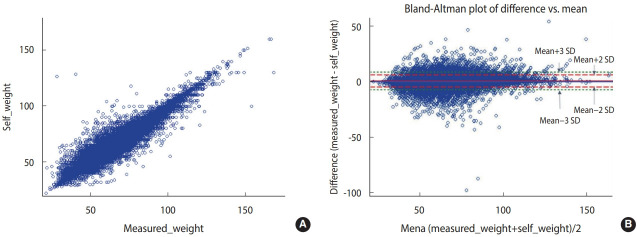
Correlation distribution and Bland-Altman plot of the measured (A) and self-reported (B) weight values.

**Figure 3.**
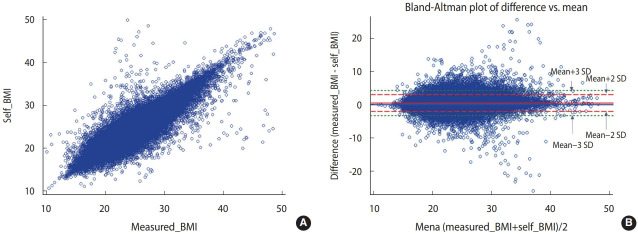
Correlation distribution and Bland-Altman plot of the measured (A) and self-reported (B) body mass index (BMI) values.

The correlation coefficients between the measured and self-reported height, weight, and BMI values were 0.96, 0.98, and 0.93, respectively, showing high correlations (Figures 1-3).

The Bland-Altman plot comparing the difference between the measured and self-reported values showed the largest difference between the two values (measured values and self-reported values) when the average self-reported height was 125-150 cm. When the average self-reported height was greater than 150 cm, the difference tended to decrease. The mean of the difference (-0.97) between the measured and self-reported values was close to 0. The 95% CI (limits of agreement) were -6.26 to 4.31 using the formula of mean± 2*standard deviation (SD) and -8.91 to 6.96 using the formula of mean± 3*SD (Figure 1). The difference between the measured and self-reported weight values was the greatest when the mean of measured and self-reported height values was 75-100 kg. When the mean of the two values was less than 75 kg or greater than 100 kg, the difference tended to decrease. The mean of the difference (0.52) between the measured and self-reported values was close to 0. The 95% CI were -4.80 to 5.84 using the formula of mean ± 2*SD and -7.50 to 8.50 using the formula of mean± 3*SD (Figure 2). The difference between the measured and self-reported BMI values was the greatest when the mean of the measured and self-reported values was 30-40. When the mean of the two values was less than 30 or greater than 40, the difference tended to decrease. The mean of the difference (0.49) between the measured and self-reported values was close to 0. The 95% CI values were -1.20 to 2.98 using the formula of mean ± 2*SD and -3.24 to 4.23 using the formula of mean± 3*SD (Figure 3).

The self-reported height values were 0.59 cm and 0.71 cm greater than the measured values for men and women, respectively. In particular, both men and women tended to report height values greater than the actual measurements as the age increased. Men aged 30-39 years, 50-59 years, and ≥ 70 years reported height values 0.26 cm, 0.65 cm, and 1.70 cm greater than the actual measured height values, respectively. Women aged 30-39 years, 50-59 years, and ≥ 70 years reported height values 0.20 cm, 0.75 cm, and 2.17 cm greater than the actual measured height values, respectively. In men aged 19-29 years, the difference between measured and self-reported values was minimal. However, in women aged 19-29 years, the self-reported height was 0.11 cm lesser than the actual measured height (Table 2).

In men and women, the self-reported weight values were 0.55 kg and 0.67 kg lesser than the actual measured weight, respectively. The difference between self-reported and measured weight values was greater in middle-aged and older participants. The difference decreased significantly in participants aged ≥ 70 years. In men aged 30-39 years, 50-59 years, and ≥ 70 years, the self-reported weight values were 0.72 kg, 0.58 kg, and 0.06 kg lesser than the actual measured weight, respectively. In women aged 30-39 years, 50-59 years, and ≥ 70 years, the self-reported weight values were 0.74 kg, 0.75 kg, and 0.37 kg lesser than the actual measured weight, respectively. In all age groups, women self-reported lower weight than men (Table 2).

The self-reported BMI values were 0.35 kg/m^2^ and 0.49 kg/m^2^ lesser than the actual measured BMI for men and women, respectively. In both men and women, the self-reported values tended to be lesser than the actual measured BMI as the age increased. In men aged 30-39 years, 50-59 years, and ≥ 70 years, the self-reported BMI values were 0.31 kg/m^2^, 0.38 kg/m^2^, and 0.49 kg/m^2^ lesser than the actual measured BMI, respectively. In women aged 30-39 years, 50-59 years, and ≥ 70 years, the self-reported BMI values were 0.34 kg/m^2^, 0.53 kg/m^2^, and 0.83 kg/m^2^ lesser than the actual measured BMI, respectively (Table 2).

### Agreement between the measured and self-reported body mass index values per category

Agreement between the measured and self-reported BMI values was analyzed by category (underweight, normal, overweight, and obesity). The kappa statistics of 0.82 and 79.6% agreement were observed. In the underweight category, a 73.8% agreement was found between the measured and self-reported BMI values. In normal, overweight, and obesity categories, the agreement rates were 81.8%, 67.3%, and 92.6%. The agreement between measured and self-reported BMI values was the highest in the obesity group. In addition, the agreement rates and kappa statistics were higher in men than in women (Table 3).

## DISCUSSION

Herein, we observed that the self-reported height and weight were over-reported and under-reported than the measured value, respectively. As a result, the self-reported BMI was underestimated than the measured BMI. In particular, men and women showed opposite findings on the difference between the measured and self-reported height values. When men reach the age of 19 years, they undergo body measurement as part of the requirements for army enlistment and are updated on their latest height. By contrast, most women only measure their height in school, leading to differences and errors between the measured and self-reported height values. Additionally, the self-reported and measured values were only evaluated simultaneously during the CHS in 2018. In particular, the self-reported obesity rate increased by 3.2%p from 28.6% in 2017 to 31.8% in 2018 [23]. This increase was approximately four times greater than the average increase of 0.9%p in the last five years from 2013, which reflects the honest responses of the participants in the self-administered questionnaires.

Consistent with our finding, in a study of 2,198 participants on the accuracy of self-reported height, weight, and BMI in the CHS, Jeong et al. [10] showed that both men and women over-reported their height, (men: 0.48 cm, women: 0.38 cm), under-reported their weight (men: -0.74 kg, women: -1.23 kg), and under-reported their BMI (men: 0.39 kg/m^2^, women: 0.60 kg/m^2^). In a comparison of the self-reported results of the 2010 CHS and measured prevalence of obesity in the 2010 KNHANES, Park et al. [24] reported that obesity and overweight were underestimated by 8.6%p and 7.8%p, respectively, in the CHS. Ki et al. [25] compared the estimates of the CHS and measured values of the KNHANES from 2010 and 2015 and observed absolute differences between the two surveys (8.9%p in 2010, 8.7%p in 2011, 8.8%p in 2012, 8.3%p in 2013, 6.6%p in 2014, and 8.2%p in 2015). Additionally, Kim et al. [26] compared the differences between measured values in the 2018 KNHANES and CHS. Although no significant difference was found, height and weight were under-reported in the CHS than in the KNHANES, while not difference was observed in BMI values between the two surveys.

Among the Organization for Economic Cooperation and Development countries, only 20, including Korea, provided both the measured and self-reported prevalence of obesity, 4 provided the measured values alone, and 13 provided the self-reported values alone. In a comparison of obesity prevalence in countries that provided the measured and self-reported values, significant differences were observed in the prevalence of obesity [26]. In Korea, the prevalence of obesity, defined as BMI of ≥ 30 kg/m^2^, were 5.9% based on the measured values and 4.3% based on the selfreported values in 2018, showing a difference of 1.6%p. Similarly, in the United States, the prevalence of obesity were 40.0% based on the measured values and 30.2% based on the self-reported values in 2016, showing a difference of 9.8%p [27].

Based on these findings, in order to develop effective methods and standardized surveys for monitoring the prevalence of obesity in Korea, considering that the CHS was only used for establishing and evaluating the Community Health Plans, accurate regional statistics are essential; take into account the survey conditions and cost-effectiveness, measurement surveys with cycles need to be conducted. In particular, external quality control by experts must be implemented for measurement surveys in order to improve the accuracy and reliability. Our findings also showed that young men who had high income, had high levels of education, and were unmarried did not undergo body measurements. Thus, in future body measurement surveys, calculating the prevalence of obesity using the height and weight values from the National Health Insurance Service health examination data may also be considered. However, this must be preceded by an amendment of laws and regulations on the personal information collected for the use of resident registration numbers and review of consent for data link agreements between institutions.

In conclusion, there was a significant difference between selfreported and measured values of the CHS. The measured values, rather than the self-reported values, were similar to the KNHANES results. This study is meaningful as it compared the self-reported and measured height and weight values of the 2018 CHS to accurately calculate the prevalence of obesity and suggest an efficient survey method. Therefore, to accurately calculate the prevalence of obesity in the CHS, BMI may be assessed using the measured height and weight values. In large-scale surveys, use of self-administered questionnaires, which are cost-effective, may lead to the underestimation of results. However, continuous monitoring using consistent standards and survey methods may help collect meaningful statistical data to compare the time series trends in the obesity rate. As the CHS provides basic regional health statistic data for establishing and evaluating Community Health Plans, the prevalence of obesity should be calculated using the measured values for use by local governments to promote community health projects based on more accurate statistical data.

## Figures and Tables

**Table 1.** Participants’ general characteristics

Characteristics	Participate in both interview and measurement^1^	Participate in interview only
Total	167,003 (73.1)	44,472 (19.5)
Gender		
Men	77,048 (46.1)	21,627 (48.6)
Women	89,955 (53.9)	22,845 (51.4)
Age (yr)		
19-29	17,285 (10.4)	5,713 (12.8)
30-39	20,582 (12.3)	7,078 (15.9)
40-49	27,271 (16.3)	9,047 (20.3)
50-59	34,052 (20.4)	9,502 (21.4)
60-69	33,110 (19.8)	7,247 (16.3)
≥70	34,703 (20.8)	5,885 (13.2)
Income (10^4^ Korean won)		
<100	24,109 (14.8)	3,705 (9.0)
100-199	26,428 (16.3)	5,462 (13.3)
200-299	25,201 (15.5)	6,440 (15.7)
300-399	24,059 (14.8)	6,723 (16.4)
400-499	18,707 (11.5)	5,220 (12.7)
500-599	19,650 (12.1)	6,050 (14.7)
≥600	24,268 (14.9)	7,489 (18.2)
Education		
Less than elementary school	37,496 (22.5)	5,633 (12.7)
Less than middle school	20,239 (12.1)	4,228 (9.5)
Less than high school	49,109 (29.4)	14,162 (32.0)
Less than college	54,460 (32.7)	18,389 (41.5)
Graduate school or higher	5,490 (3.3)	1,889 (4.3)
Occupation		
Management‧profession	17,236 (10.3)	5,872 (13.2)
Office worker	14,657 (8.8)	5,314 (12.0)
Sales‧service	20,882 (12.5)	6,563 (14.8)
Agriculture‧forestry‧fishing	19,120 (11.5)	3,353 (7.6)
Manipulation of technical‧devices	15,983 (9.6)	4,596 (10.4)
Simple labor	16,140 (9.7)	3,420 (7.7)
Soldier	574 (0.3)	154 (0.3)
Others (housewife, student, or no occupation)	62,246 (37.3)	15,074 (34.0)
Marital status		
Married (with spouse)	114,710 (68.7)	30,187 (68.1)
Other (widow, divorce, etc.)	27,079 (16.2)	5,466 (12.3)
Unmarried	25,062 (15.0)	8,671 (19.6)
Area		
Urban	95,776 (57.3)	28,877 (64.9)
Rural	71,227 (42.7)	15,595 (35.1)
Generation type		
1st generation	78,933 (47.3)	17,967 (40.4)
2nd generation	76,396 (45.7)	23,029 (51.8)
3rd generation and over	11,671 (7.0)	3,441 (7.7)
Self-reported obesity		
Obesity (25.0 ≤BMI)	51,135 (30.7)	14,224 (32.0)^1^
Normal (18.5≤BMI<25.0)	108,845 (65.2)	28,444 (64.0)^1^
Underweight (BMI<18.5)	6,852 (4.1)	1,786 (4.0)^1^

Values are presented as number (%). KRW, Korean won; BMI, body mass index.

1 Participants who provided both self-reported and measured values, including those who had outlier values.

**Table 2.** Comparison of self-reported and measured height, weight, and BMI values

Variables		Self-reported		Measured		Difference^1^	
		n	Mean±SE	n	Mean±SE	Mean (95% CI)	p-value
Height (cm)							
	Men (yr)						
	Total	78,260	171.62±2.94	78,260	171.03±3.13	-0.59 (-0.61, -0.57)	<0.001
	19-29	8,515	174.49±7.02	8,515	174.46±7.15	-0.03 (-0.07, 0.00)	0.068
	30-39	9,866	174.78±6.29	9,866	174.52±6.51	-0.26 (-0.30, -0.22)	<0.001
	40-49	13,016	172.76±5.85	13,016	172.33±6.03	-0.43 (-0.47, -0.39)	<0.001
	50-59	15,459	170.32±5.47	15,459	169.67±5.67	-0.65 (-0.68, -0.62)	<0.001
	60-69	15,420	168.16±5.83	15,420	167.14±6.09	-1.02 (-1.06, -0.98)	<0.001
	≥70	15,984	166.26±6.30	15,984	164.55±6.48	-1.70 (-1.77, -1.64)	<0.001
	Women (yr)						
	Total	93,124	158.58±2.64	93,124	157.87±2.82	-0.71 (-0.73, -0.69)	<0.001
	19-29	9,437	161.51±6.23	9,437	161.62±6.33	0.11 (0.08, 0.14)	<0.001
	30-39	11,462	161.63±5.54	11,462	161.43±5.65	-0.20 (-0.22, -0.17)	<0.001
	40-49	15,054	159.98±5.10	15,054	159.59±5.22	-0.39 (-0.42, -0.36)	<0.001
	50-59	19,276	157.79±4.60	19,276	157.04±4.80	-0.75 (-0.78, -0.72)	<0.001
	60-69	18,336	155.89±4.91	18,336	154.59±5.11	-1.30 (-1.35, -1.26)	<0.001
	≥70	19,559	153.08±6.01	19,559	150.92±5.79	-2.17 (-2.24, -2.10)	<0.001
Weight (kg)							
	Men (yr)						
	Total	80,502	71.96±5.44	80,502	72.52±5.65	0.55 (0.53, 0.58)	<0.001
	19-29	8,584	73.63±16.70	8,584	74.22±17.36	0.59 (0.53, 0.65)	<0.001
	30-39	10,025	77.08±12.94	10,025	77.80±13.37	0.72 (0.67, 0.78)	<0.001
	40-49	13,236	74.17±11.15	13,236	74.83±11.44	0.67 (0.62, 0.71)	<0.001
	50-59	15,788	70.86±9.75	15,788	71.44±10.19	0.58 (0.53, 0.62)	<0.001
	60-69	15,833	67.95±9.47	15,833	68.46±9.81	0.51 (0.45, 0.56)	<0.001
	≥70	17,036	64.23±9.78	17,036	64.30±10.26	0.06 (0.00, 0.13)	0.069
	Women (yr)						
	Total	97,337	57.28±3.78	97,337	57.95±3.97	0.67 (0.65, 0.69)	<0.001
	19-29	9,106	55.92±11.82	9,106	56.58±12.80	0.67 (0.61, 0.72)	<0.001
	30-39	11,086	57.81±10.00	11,086	58.55±10.49	0.74 (0.70, 0.78)	<0.001
	40-49	14,679	57.87±8.54	14,679	58.65±8.95	0.78 (0.74, 0.82)	<0.001
	50-59	19,377	58.21±7.24	19,377	58.96±7.62	0.75 (0.72, 0.79)	<0.001
	60-69	19,179	58.07±7.82	19,179	58.71±8.18	0.64 (0.60, 0.69)	<0.001
	≥70	23,910	55.37±7.71	23,910	55.74±7.96	0.37 (0.32, 0.42)	<0.001
BMI (kg/m^2^)							
	Men (yr)						
	Total	76,771	24.38±1.59	76,771	24.73±1.68	0.35 (0.35, 0.36)	<0.001
	19-29	8,321	24.11±5.06	8,321	24.31±5.28	0.20 (0.18, 0.22)	<0.001
	30-39	9,670	25.16±3.79	9,670	25.47±3.96	0.31 (0.29, 0.33)	<0.001
	40-49	12,812	24.81±3.35	12,812	25.16±3.47	0.35 (0.33, 0.37)	<0.001
	50-59	15,217	24.39±2.90	15,217	24.77±3.07	0.38 (0.37, 0.40)	<0.001
	60-69	15,158	23.99±2.90	15,158	24.47±3.06	0.48 (0.45, 0.50)	<0.001
	≥70	15,593	23.25±3.22	15,593	23.74±3.34	0.49 (0.46, 0.52)	<0.001
	Women (yr)						
	Total	89,649	22.84±1.50	89,649	23.33±1.60	0.49 (0.48, 0.50)	<0.001
	19-29	8,912	21.44±4.23	8,912	21.66±4.42	0.22 (0.20, 0.24)	<0.001
	30-39	10,843	22.13±3.68	10,843	22.47±3.88	0.34 (0.32, 0.36)	<0.001
	40-49	14,370	22.63±3.23	14,370	23.05±3.39	0.42 (0.40, 0.44)	<0.001
	50-59	18,725	23.38±2.72	18,725	23.91±2.92	0.53 (0.52, 0.55)	<0.001
	60-69	17,837	23.90±3.12	17,837	24.58±3.33	0.68 (0.65, 0.70)	<0.001
	≥70	18,962	23.77±3.22	18,962	24.60±3.35	0.83 (0.80, 0.87)	<0.001

BMI, body mass index; SE, standard error; CI, confidence interval.

1 Difference=measured value minus self-reported value.

**Table 3.** Agreement between measured and self-reported body mass index (BMI) values per category

Measured BMI		Self-reported BMI^1^					Agreement (%)	Kappa (p-value)
		Underweight	Normal	Overweight	Obesity	Total		
Total		6,836 (100)	67,439 (100)	41,186 (100)	50,959 (100)	166,420	79.6	0.82 (<0.001)
	Underweight	5,047 (73.8)	1,765 (2.6)	37 (0.1)	17 (0.0)	6,866		
	Normal	1,703 (24.9)	55,158 (81.8)	3,991 (9.7)	493 (1.0)	61,345		
	Overweight	51 (0.7)	9,210 (13.7)	27,736 (67.3)	3,280 (6.4)	40,277		
	Obesity	35 (0.5)	1,306 (1.9)	9,422 (22.9)	47,169 (92.6)	57,932		
Men		1,983 (100)	25,600 (100)	21,315 (100)	27,873 (100)	76,771	80.5	0.82 (<0.001)
	Underweight	1,403 (70.8)	557 (2.2)	20 (0.1)	7 (0.0)	1,987		
	Normal	554 (27.9)	19,665 (76.8)	1,411 (6.6)	196 (0.7)	21,826		
	Overweight	14 (0.7)	4,880 (19.1)	14,500 (68.0)	1,438 (5.2)	20,832		
	Obesity	12 (0.6)	498 (1.9)	5,384 (25.3)	26,232 (94.1)	32,126		
Women		4,853 (100)	41,839 (100)	19,871 (100)	23,086 (100)	89,649	78.8	0.80 (<0.001)
	Underweight	3,229 (66.5)	657 (1.6)	11 (0.1)	8 (0.0)	3,905		
	Normal	1,549 (31.9)	33,437 (79.9)	1,333 (6.7)	232 (1.0)	36,551		
	Overweight	48 (1.0)	6,643 (15.9)	12,184 (61.3)	1,069 (4.6)	19,944		
	Obesity	27 (0.6)	1,102 (2.6)	6,343 (31.9)	21,777 (94.3)	29,249		

Values are presented as number (%).

1 Underweight (<18.5 kg/m^2^), normal (18.5-22.9 kg/m^2^), overweight (23.0-24.9 kg/m^2^), and obesity (≥25.0 kg/m^2^).

## SUPPLEMENTARY MATERIALS

Korean version is available at https://www.e-epih.org/.
