# Supplementary material for: Differences in accuracy of height, weight, and body mass index between self-reported and measured using the 2018 Korea Community Health Survey data
Source: Epidemiol Health. 2022 Feb 19;44:e2022024. doi: 10.4178/epih.e2022024 (PMC9117107; doi:10.4178/epih.e2022024)

**Title:**

**(국문) 자가보고와 실제 측정 간 신장, 체중, 체질량지수의 정확성 차이: 2018년 지역사회건강조사 결과**

**(영문) Differences in accuracy of height, weight, and BMI between self-reported and measured**

**Running title**: Differences in accuracy of BMI

**Authors:**

고윤실^1^, 최선혜^1^, 원지수^1^, 이연경^2^, 김동현^3^, 이선규^1^

Yoonsil Ko^1^, Sunhye Choi^1^, Jisoo Won^1^, Yeon-Kyeng Lee^2^, Dong-Hyun Kim^3^, and Seon Kui Lee^1^

**Affiliations:**

^1^질병관리청 만성질환관리국 만성질환관리과, ^2^질병관리청 의료안전예방국 의료감염관리과, ^3^한림대학교 의과대학

^1^ Division of Chronic Disease Control, Bureau of Chronic Disease Prevention and Control, Korea Disease Control and Prevention Agency, Cheongju, Korea; ^2^ Division of Healthcare Association Infection Control, Bureau of Healthcare Safety and Immunization, Korea Disease Control and Prevention Agency, Cheongju, Korea; ^3^ Department of Social and Preventive Medicine, Hallym University College of Medicine, Graduate School of Public Health

**Corresponding author:** Seon Kui Lee, PhD.

Division of Chronic Disease Control, Bureau of Chronic Disease Prevention and Control, Korea Disease Control and Prevention Agency, Osong Health Technology Administration Complex, 187 Osongsaengmyeong 2-ro, Osong-eup, Heungduk-gu, Cheongju-si, Chungcheongbuk-do, 28159 Republic of Korea

Tel: 043-719-7430

E-mail: byuly74@korea.kr

**ABSTRACT**

**목적**: 이 연구의 목적은 지역사회건강조사 2018년 조사자료를 통해 자가보고된 신장, 체중, 그리고 체질량지수와 실제 측정된 값을 비교하여 정확한 비만유병률을 산출하기 위한 조사방법을 확인하는 것이다.

**방법**: 2018년 지역사회건강조사 원시자료를 이용하여, 자가보고된 신장, 체중, 그리고 체질량지수 값과 실제 측정한 값 간에 차이를 분석하고 상관성과 일치도를 확인하고자 한다.

**결과**: 본 연구결과 자가보고로 조사된 신장은 측정값보다 과대보고(남자는 측정값보다 자가보고값이 0.59cm, 여자는 0.71cm 크게 보고)되고 체중은 과소보고(남자는 측정값보다 자가보고값이 0.55kg, 여자는 0.67kg 적게 보고)되었음을 확인하였다. 이에 따라 자가보고값 체질량지수는 측정값보다 과소측정(남자의 경우 측정값보다 자가보고값이 0.35kg/m^2^ 낮게, 여자는 0.49kg/m^2^ 낮게 측정)된다는 것을 확인할 수 있었다. 측정값과 자가보고값 체질량지수 구간별(저체중, 정상, 과체중, 비만) 일치도는 79.6%, 카파값은 0.82로 나타났다.

**결론**: 정확한 근거자료를 기반으로 지역보건사업을 추진할 수 있도록 지역사회건강조사에서 실제 측정을 통한 비만유병률을 산출하는 것이 필요하겠다.

**주제어**: 지역사회건강조사, 비만율, 측정, 자가보고

**INTRODUCTION**

과체중과 비만은 건강을 해칠 수 있는 비정상적인 또는 과도한 지방의 축적으로 정의한다(1). 비만을 측정하기 위한 간접적인 방법으로 체중을 신장의 제곱으로 나눈 값(kg/m^2^)인 체질량 지수(Body Mass Index, BMI)를 성인의 과체중(25kg/m^2^ 이상)과 비만(30kg/m^2^ 이상) 분류에 일반적으로 사용한다(1). 전 세계적으로 2016년 기준 18세 이상 성인 약 19억 명 이상이 과체중이었고, 이 중 6억 5천만 명 이상이 비만으로 세계 성인 인구의 약 13%(남성 11%, 여성 15%)를 차지하였으며 1975년 이후 거의 3배 수준으로 나타났다(1). 우리나라는 2019년 기준 성인의 비만유병률(BMI 25kg/m^2^ 이상)은 33.8%(만 19세 이상, 표준화)로 지속 증가추세를 보이고 있다(2). 비만은 제2형 당뇨병, 심혈관질환, 고혈압, 뇌졸중 및 각종 암 등 비감염성 질환의 주요 위험요소이며 동반질환으로 인한 사회경제적 부담이 증가되고 삶의 질도 저하된다고 하였다(3). 이러한 문제로 WHO는 2004년 제57차 연차총회에서 ‘식이, 신체활동과 건강에 대한 전 세계적 전략’을 발표하였으며, 전 세계적으로 문제가 되는 비만을 예방, 관리하기 위해 끊임없이 노력하고 있다(4). 비만은 예방 가능한 질병이므로(1), 정확한 비만유병률을 파악하고 이에 맞는 효과적인 예방관리를 위한 지속적인 정책 마련이 요구된다.

우리나라는 국민건강영양조사와 지역사회건강조사를 통해 국가 및 지역단위 비만유병률을 파악하고 있으며 비만을 예방하고 관리하기 위한 국가 및 지방자치단체의 정책 수립 및 평가 시 근거자료로 활용하고 있다. 국민건강영양조사는 조사대상 약 1만 명에 대해 신장과 체중을 실제 계측하여 비만유병률을 산출하며, 지역사회건강조사는 약 23만 명을 대상으로 하여 편의성과 경제성 때문에 2008년부터 자가보고로 비만율을 조사하였으나 2018년부터 신체계측조사를 도입하여 비만유병률을 산출하기 시작하였다.

많은 선행연구에 따르면 자가보고에 기반한 BMI 측정은 여러 대규모 역학조사에서 상대적 편의성과 효율성으로 인해 실제 계측을 대신하여 사용하고 있으나 자가보고 값은 대상자에 따라 체중은 과소보고 되고 신장은 과대 보고되는 오류가 발생하여 부정확하다는 연구결과가 나타났으며(5-10), 일부 연구에서는 자가보고 값과 실측값 간의 상관계수가 0.8이상 높은 수준을 보인다는 결과도 있었다(10-19). 국내 연구에서도 자가보고 신장과 체중의 정확성 연구가 수행된 바 있고, 비만도 평가를 위해서는 실측치를 사용할 것을 권고하였으며(20), 가급적 신장, 체중 그리고 비만율 등은 실측자료에 기반하여야 한다고 하였다(10). 또한 자가보고 값이 임상 및 공중보건 현장에서 사용할 정도의 정확성을 가지고 있다는 보고도 있었지만 병원 검진센터 방문자 또는 입원환자 대상 연구라는 제한점이 있었다(19, 21).

이에 본 연구는 실제 측정과 자가보고 신장과 체중을 동시 조사한 2018년 지역사회건강조사 자료를 활용하여 2가지 방법으로 조사한 결과 값의 비교분석을 통해 보다 정확한 비만유병률을 산출하기 위한 조사방법을 확인하고자 한다.

**SUBJECTS AND METHODS**

본 연구는 2018년 지역사회건강조사 원시자료를 사용하였다. 지역사회건강조사는 전국 254개 보건소가 지역주민의 건강실태 파악을 위해 보건소당 약 900명의 표본(만 19세 이상)을 대상으로 매년 8~10월 가구방문 면접조사로 실시한다. 2018년 조사참여자는 228,340명이며, 이 중 ‘응답거부’ 또는 ‘모름’으로 응답한 사례와 이상치(신장 50cm 미만, 200cm 초과, 체중 20kg 미만, 130kg 초과, BMI 10kg/m^2^ 미만, 50kg/m^2^ 초과)에 해당하는 경우를 제외하여 최종 설문응답자 214,640명과 신체계측 측정자 183,211명을 대상으로 분석하였다. 신체계측 제외대상은 서있을 수 없거나 임신한 여성으로 조사 시 제외하였다.

2018년 지역사회건강조사는 자가기입과 신체계측 신장과 체중을 동시에 조사한 유일한 시기이며, 실제 계측 전 사전설문으로 ‘평소 알고 계시는 신장과 체중은 얼마입니까?’를 질문하였다.

신체계측 측정도구는 가구방문 면접조사 특성 상, 휴대가 편리하고 측정오차범위가 크지 않은 도구를 선정하여 신장계는 인바디사의 초음파 신장계인 InLabS50, 체중계는 CAS사의 HE-58을 도입하였다. 본 측정도구를 이용한 신장은 선 키를 기준으로 총 2회 측정하여 평균값을 사용하였고, 체중은 1회 측정한 값을 사용하였다. 초음파 신장계의 경우 성인의 키 측정의 신뢰도와 유효성을 검증한 바 있다(22).

통계분석은 SAS 9.4 프로그램을 이용하였고 복합표본설계를 고려하여 성별, 연령군별(19-29세, 30-39세, 40-49세, 50-59세, 60-69세, 70세 이상)로 층화 분석하였으며 통계적 유의수준은 0.05 미만으로 하였다. 분석방법은 신장, 체중, BMI의 자가보고와 측정 값의 차이를 확인하기 위해 paired t-test를 사용하여 평균과 95% 신뢰구간(confidence interval, CI)을 산출하였다. 또한 두 값 간의 상관성을 Pearson correlation으로 분석하고 측정값과 자가보고값 간 차이가 있는지 확인하고자 Bland-Altman 분석을 추가하였다. BMI군별 일치도 평가를 위해 자가보고 및 측정값을 기반으로 한 4개 군(<18.5, 18.5-22.9, 23-24.9, ≥25kg/m^2^)으로 범주화하여 카파통계량(kappa statistic)을 확인하였다. 분석 시 측정값은 예비조사를 통해 확인된 초음파 신장계의 측정오차를 보정하고자 신장의 경우 측정값에서 0.4cm를 뺀 값을 사용하였다.

**RESULTS**

**대상자의 일반적 특성**

지역사회건강조사 참여대상자 중 본 연구에 활용된 자가보고 설문과 신체계측을 모두 참여한 대상자와 자가보고 설문은 참여하였으나 신체계측은 미참여한 대상자 간 특성을 비교하였다. 결과를 살펴보면 남자, 젊은 연령대, 고소득층, 고학력층, 전문직 또는 사무직 종사자, 미혼자, 동 지역거주자, 2세대인 가구유형, 비만(자가보고 기준)일 경우 자가보고 설문만 참여하고 신체계측에 미참여한 경우가 많았으며, 이는 통계적으로 유의한 결과를 보였다(표1).

**신장, 체중, BMI의 측정값과 자가보고값의 비교**

측정값과 자가보고값의 상관분포도를 살펴보면, 신장의 측정값과 자가보고값 간의 상관계수는 0.96, 체중의 측정값과 자가보고값 간의 상관계수는 0.98, 측정값으로 산출된 BMI와 자가보고값으로 산출된 BMI 간의 상관계수는 0.93으로 매우 높게 나타났다(그림1~3). 또한 측정값과 자가보고값 간의 차이를 비교한 Bland-Altman plot 결과에서는, 신장의 경우 자가보고값과 측정값의 평균이 125~150cm 구간에 있을 때 두 측정값 간의 차이(계측값-자가보고값)가 가장 많이 나는 양상을 보였으며, 측정값 간 평균이 150cm 보다 커질수록 차이는 점점 줄어드는 양상이 확인되었다. 측정값 간 차이의 평균(-0.97)은 0에 가까웠으며, 95% 신뢰구간(LOA)은 평균±2*표준편차의 산식으로 하면 -6.26, 4.31 범위이며 동 산식에 3*표준편차를 적용한 경우 -8.91, 6.96로 나타났다(그림1). 체중은 자가보고값과 측정값의 평균이 75~100kg 구간에 있을 때 두 측정값 간 가장 많은 차이가 났으며, 측정값 간 평균이 75kg미만 또는 100kg 초과할수록 차이는 점점 줄어들었다. 측정값 간 차이의 평균(0.52)은 0에 매우 가까웠고, 95% 신뢰구간은 평균±2*표준편차 적용 시 -4.80, 5.84이며, 3*표준편차를 적용하면 -7.50, 8.50이었다(그림2). 체질량지수는 자가보고값과 측정값의 평균이 30~40 사이 구간에서 두 측정값 간 가장 많은 차이가 났고, 측정값 간 평균이 30미만 또는 40을 초과할수록 차이는 점점 줄어드는 것을 확인할 수 있었다. 두 측정값 간 차이의 평균(0.49)은 0에 근접하였으며, 95% 신뢰구간은 평균±2*표준편차 기준으로는 -1.20, 2.98이었고 3*표준편차를 적용하면 -3.24, 4.23로 나타났다(그림3).

측정값과 자가보고값을 비교해보면, 우선 신장의 경우 남자는 측정값보다 자가보고값이 0.59cm, 여자는 0.71cm 크게 보고한 것으로 나타났다. 특히 남녀 모두 연령이 높을수록 측정값보다 크게 보고하는 경향이 관찰되었는데, 남자의 경우 30-39세에서 측정값보다 0.26cm, 50-59세는 0.65cm, 70세 이상에서는 1.7cm 크게 보고하였으며, 여자는 30-39세에서 측정값보다 0.2cm, 50-59세는 0.75cm, 70세 이상에서는 2.17cm 크게 보고한 것으로 나타났다. 한편 19-29세는 남자의 경우 측정값과 자가보고값 간에 차이가 거의 없었으나, 여자는 오히려 실제보다 0.11cm 작게 보고한 것으로 나타났다(표2). 체중의 경우 남자는 측정값보다 자가보고값이 0.55kg, 여자는 0.67kg 적게 보고한 것으로 나타났다. 남녀 모두 중장년층에서 측정값과 자가보고값의 차이가 더 크게 났으며 70세 이상에서는 차이가 현저하게 줄어드는 것을 확인할 수 있었다. 남자의 경우 측정값보다 30-39세에서 0.72kg, 50-59세는 0.58kg, 70세 이상에서는 0.06kg 적게 보고하였고, 여자는 30-39세에서 0.74kg, 50-59세는 0.75kg, 70세 이상에서는 0.37kg 적게 보고한 것으로 나타났다. 체중은 모든 연령대에서 여자가 남자보다 더 적게 보고한 것으로 나타났다(표2). 다음으로 BMI를 살펴보면, BMI는 남자의 경우 측정값보다 자가보고값이 0.35kg/m^2^ 낮게, 여자는 0.49kg/m^2^ 낮게 나타났다. 특히 남녀 모두 연령이 높을수록 자가보고값 BMI가 측정값 BMI 보다 작아지는 경향을 보였는데, 남자의 경우 30-39세는 측정값보다 자가보고값이 0.31kg/m^2^, 50-59세는 0.38kg/m^2^, 70세 이상에서는 0.49kg/m^2^ 작게 나타났으며, 여자는 30-39세에서 0.34kg/m^2^, 50-59세는 0.53kg/m^2^, 70세 이상에서는 0.83kg/m^2^ 작게 나타났다(표2).

**측정값과 자가보고값의 BMI 구간별 일치도**

측정값을 기준으로 산출된 BMI와 자가보고값을 기준으로 산출된 BMI를 이용하여 각각의 BMI 구간별(저체중, 정상, 과체중, 비만) 일치도를 측정한 결과 79.6%의 일치도와 0.82의 카파값이 확인되었다. 저체중 구간에서는 대상자의 73.8%에서 실측값과 자가보고의 값이 일치하였고 정상 구간에서는 81.8%, 과체중 구간에서는 67.3%, 비만 구간에서는 92.6%에서 측정값과 자가보고의 값이 일치하였으며, 비만 그룹에서 일치도가 가장 높게 나타났다. 또한, 여자보다는 남자에서 일치도와 카파값이 더 높았다(표3).

**Discussion**

본 연구결과 자가보고로 조사된 신장은 측정값보다 과대보고되고 체중은 과소보고되었음을 확인하였다. 이에 따라 자가보고값 BMI는 측정값보다 과소측정된다는 것을 확인할 수 있었다. 특히 신장의 경우 20대 남자와 여자의 측정값과 자가보고값 간 차이가 상반된 결과를 보이는데, 이는 남자의 경우 만 19세가 되면 징병검사를 통해 신장을 측정하여 가장 최신 신장을 알고 있는 반면, 여자는 신장을 측정한 시기가 학창시절인 경우가 대부분이어서 측정시점 차이로 인해 나타난 결과로 보여진다. 또한 2018년은 지역사회건강조사에서 신체계측조사와 자가보고 설문 조사를 동시에 실시한 유일한 해이며, 특히 신체계측 직전에 평소 본인이 알고 있는 신장과 체중을 확인하여 2017년 자가보고 비만율 28.6%에 비해 2018년은 31.8%로 3.2%p 증가하였다(23). 2013년부터 5년 간 증가율 평균 0.9%p에 비해 약 4배 정도 높게 나타나 이는 실측을 앞두고 자가보고 설문에 솔직하게 응답한 결과로 보여진다.

본 연구와 유사한 선행연구를 살펴보면, 정진영 등(2016)은 지역사회건강조사의 자가보고 신장, 체중 그리고 체질량지수의 정확성을 확인하고자 2016년 부가적으로 2,198명을 조사하여 남녀 모두 신장은 실제보다 크게(남자 0.48cm, 여자 0.38cm), 체중은 가볍게(남자 –0.74kg, 여자 –1.23kg), 그리고 BMI는 남녀 각각 0.39kg/m^2^, 0.60kg/m^2^ 과소보고 되었다(10). 박여린 등(2013)은 2010년 지역사회건강조사 자가보고 결과와 2010년 국민건강영양조사의 실측한 비만유병률 결과를 비교했을 때 지역사회건강조사 결과가 비만은 8.6%p, 과체중은 7.8%p 저평가된다고 확인하였으며(24), 기지선 등(2019)은 2010년부터 2015년까지 지역사회건강조사 설문 추정치와 국민건강영양조사 검진 추정치를 비교한 결과, 두 조사의 절대적 차이가 있는 것을 확인하였다(2010년 8.9%p, 2011년 8.7%p., 2012년 8.8%p, 2013년 8.3%p, 2014년 6.6%p, 2015년 8.2%p)(25). 김승현 등(2020)은 2018년 지역사회건강조사와 국민건강영양조사 실측값 간의 차이를 확인한 결과 신장과 체중이 통계적으로 유의하진 않았지만 지역사회건강조사가 국민건강영양조사보다 작게 측정되었다고 확인하였고 BMI는 두 조사 간 차이가 유의하지 않은 것으로 나타났다(26). 또한 OECD 국가별 비만유병률을 살펴보면, 측정한 결과와 자가보고한 결과를 모두 제공하는 국가는 우리나라를 포함하여 20개국이고, 측정값만 제공하는 국가는 4개국, 자가보고값만 제공하는 국가는 13개국이었다. 두가지 측정 결과를 모두 제공한 국가의 비만유병률을 비교해보면 차이가 나는 것을 확인할 수 있었다(26). 우리나라도 BMI 30kg/m2이상을 기준으로 비만유병률 측정값은 2018년 기준 5.9%, 자가보고값은 4.3%로 1.6%p 차이가 있었으며, 미국도 측정값의 경우 2016년 기준 40.0%, 자가보고값은 30.2%로 9.8%p의 차이가 나는 것을 확인할 수 있었다(27).

이러한 결과를 바탕으로 우리나라 비만유병률 모니터링 시 효과적인 조사방법과 표준화된 조사수행을 위해, 특히 지역사회건강조사는 지역보건의료계획 수립 및 평가 시 근거자료로 활용된다는 점을 감안할 때, 정확한 지역별 통계생산은 필요하나 조사 여건과 비용효과적인 면을 고려하여 순환주기를 적용한 계측조사를 실시할 필요가 있겠다. 특히 계측조사 수행 시에는 정확도 및 신뢰도 향상을 위해 전문가를 통한 외부 질 관리가 추가 수행되어야 할 것이다. 추가로 신체계측에 미참여한 대상자의 특성을 보면 남자, 젊은 연령대, 고소득자, 고학력자, 미혼자 등으로 대부분 조사에 비협조적인 집단으로 나타났다. 이러한 특성을 고려하여 향후 신체계측 조사를 위해서는 국민건강보험공단 건강검진 자료 연계를 통해 신장, 체중 값을 활용하여 비만유병률을 산출하는 방안도 고려할 수 있겠다. 하지만 이를 위해서는 주민번호수집이 필요하여 개인정보 수집을 위한 법령 개정이 우선되어야 하고, 추가적으로 타 기관 자료연계 동의서 작성 등에 대해 검토가 필요하다.

**Conclusion**

결론적으로 지역사회건강조사의 자가보고값과 측정값 간에는 서로 유의한 차이가 있으며, 국민건강영양조사 결과와는 자가보고값보다 측정값이 유사한 것으로 나타났다. 본 연구는 2018년 지역사회건강조사 신체계측과 자가보고로 동시에 조사한 신장과 체중 결과를 비교하여 정확한 비만유병률 산출과 효율적인 조사수행방법을 제안하는데 의의가 있다. 따라서 지역사회건강조사도 지역단위의 정확한 비만유병률 산출을 위해서는 실제 측정을 통한 신장과 체중을 확인하여 체질량 지수를 파악하는 것이 보다 신뢰도 높은 조사방법이라 할 수 있겠다. 추가로 대규모 조사체계에서 비용효과적으로 실시할 수 있는 자가보고 설문조사도 측정값에 비해 과소보고된다는 제한점은 있으나 일관된 기준과 조사방법을 통해 모니터링을 지속해 나간다면 비만율 증감을 확인하기 위한 시계열 추이를 비교하는데에는 의미있는 통계자료로 활용될 수 있을 것이다. 그러나 지역사회건강조사는 지역단위 건강통계 생산을 통해 지역보건의료계획 수립 및 평가 시 근거자료로 제공하고 있으므로 이를 활용하는 지자체가 보다 정확한 통계자료를 근거로 지역보건사업을 추진할 수 있기 위해서는 실제 측정을 통한 비만유병률을 산출하여 기초자료로 제공하는 것이 필요하겠다.

**CONFLICT OF INTEREST**

모든 저자는 본 연구에 대해 표명할 이해상충이 없음

**FUNDING**

없음

**ACKNOWLEDGEMENTS**

지역사회건강조사에 참여해주신 대상자께 감사드립니다. 또한, 조사 수행과 지원을 하신 17개 시도 및 254개 보건소와 35개 책임대학교에도 감사드립니다.

**AUTHOR CONTRIBUTIONS**

Conceptualization: YL, YK, SC, JW. Data curation: JW, YK. Formal analysis: YK. Funding acquisition: None. Project administration: DK, YL, SL. Visualization: YK, SC. Writing – original draft: SC, YK, JW. Writing – review & editing: SC, YL, DK, SL.

**ORCID**

Yoonsil Ko: https://orcid.org/0000-0002-1441-5472; Sunhye Choi: https://orcid.org/0000-0002-2942-0290; Jisoo Won: https://orcid.org/0000-0003-2361-8666; Yeon-Kyeon Lee: https://orcid.org/0000-0002-0828-455X; Dong-Hyun Kim: https://orcid.org/0000-0002-1492-5253; Seon Kui Lee: https://orcid.org/0000-0001-6629-5591

**REFERENCES**

1. World Health Organization. Available from: https://www.who.int/news-room/fact-sheets/detail/obesity-and-overweight

2. Korea Disease Control and Prevention Agency. Korea Health Statistics 2019: Korea National Health and Nutrition Examination Survey (KNHANES Ⅷ-1). 2020.

3. Korean Society for the Study of Obesity. Available from: http://general.kosso.or.kr/html/?pmode=obesityDisease

4. World Health Organization. Global Strategy on Diet, Physical Activity and Health. 2004.

5. Lu S, Su J, Xiang Q, Zhou J, Wu M. Accuracy of self-reported height, weight, and waist circumference in a general adult Chinese population. Popul Health Metr 2016;11:30.

6. Gildner TE, Barrett TM, Liebert MA, Kowal P, Snodgrass JJ. Does BMI generated by self-reported height and weight measure up in older adults from middle-income countries? Results from the study on global AGEing and adult health (SAGE). BMC Obes 2015;26:44.

7. Yong V, Saito Y. How accurate are self-reported height, weight, and BMI among community-dwelling elderly Japanese: Evidence from a national population-based study. Geriatr Gerontol Int 2012;12:247–256.

8. Babiarczyk B, Sternal D. Accuracy of self-reported and measured anthropometric data in the inpatient population. Int J Nurs Pract 2015; 21:813–819.

9. Lois K, Kumar S, Williams N, Birrell L. Can self-reported height and weight be relied upon. Occup Med 2011;61:590–592.

10. Jung JY, Kim DH, Kim KY, Ryu SY, Lee SY, Park YS. Accuracy of self-reported height, weight and body mass index in community health survey in South. J Health Info Stat 2017;42:241–249

11. Lin CJ, DeRoo LA, Jacobs SR, Sandler DP. Accuracy and reliability of self-reported weight and height in the Sister Study. Public Health Nutr 2012;15:989–999.

12. Burton NW, Brown W, Dobson A. Accuracy of body mass index estimated from self-reported height and weight in mid-aged Australian women. Aust N Z J Public Health 2010;34:620–623.

13. Dekkers JC, van Wier MF, Hendriksen IJ, Twisk JW, van Mechelen W. Comparative effectiveness of lifestyle interventions on cardiovascular risk factors among a Dutch overweight working population: a randomized controlled trial. BMC Public Health 2011;11:49.

14. Poston WS, Jitnarin N, Haddock CK, Jahnke SA, Day RS. Accuracy of self-reported weight, height and BMI in US firefighters. Occup Med 2014;64:246–254.

15. Yoong SL, Carey ML, D’Este C, Sanson-Fisher RW. Agreement between self-reported and measured weight and height collected in general practice patients: a prospective study. BMC Med Res Methodol 2013;13:38.

16. Xie YJ, Ho SC, Liu ZM, Hui SS. Comparisons of measured and self-reported anthropometric variables and blood pressure in a sample of Hong Kong female nurses. PLoS One 2014;9:e107233.

17. Tang W, Aggarwal A, Moudon AV, Drewnowski A. Self-reported and measured weights and heights among adults in Seattle and King County. BMC Obes 2016;3:11.

18. Skeie G, Mode N, Henningsen M, Borch KB. Validity of self-reported body mass index among middle-aged participants in the Norwegian Women and Cancer study. Clin Epidemiol 2015;7:313–323.

19. Lee DH, Shin A, Kim J, Yoo KY, Sung J. Validity of self-reported height and weight in a Korean population. J Epidemiol 2011;21:30–36.

20. Song YM, Yoon JL. The accuracy of self-reported weight and height. Korean J Epidemiol 1995;17:257–268 (Korean).

21. Kim NY, Shin MH, Nam SJ, Yang JH. Validity of self-reported weight, height and body mass index in a hospital based breast cancer case-control study. Korean J Health Promot Dis Prev 2004;4:45–51 (Korean).

22. Cho SH, Cho YG, Park HA, Bong AR. Reliability and validity of an ultrasonic device for measuring height in adults. Korean J Fam Med 2020;42:376–381.

23. Korea Disease Control and Prevention Agency. Community Health Statistics 2020 Ⅰ: Summary. 2021.

24. Park YR, Cho YG, Kang JH, Park HA, Kim KW, Hur YI, et al. Comparison of obesity and overweight prevalence among Korean adults according to Community Health Survey and Korea National Health and Nutrition Examination Survey. Korean J Obes 2014;23;64–68 (Korean).

25. Ki JS. Comparison of estimates and time series stability of Korea Community Health Survey and Korea National Health and Nutrition Examination Survey [dissertation]. Korea: Seoul National University; 2018.

26. Kim SH, Pack ES. Differences in height, weight, BMI, and obesity rate between 2018 Community Health and Korea National Health and Nutrition Examination Surveys. J Health Info Stat 2020;45:281–287.

27. Organisation for Economic Co-operation and Development. Available from: https://stats.oecd.org/Index.aspx?DataSetCode=HEALTH_LVNG

**Table 1. Participants’ general characteristics**

|  | Participate in both interview and measurement | | Participate in interview only | |
| --- | --- | --- | --- | --- |
|  | N* | % | N | % |
| **Total** | 167,003 | 73.1 | 44,472 | 19.5 |
|  |  |  |  |  |
| **Sex** |  |  |  |  |
| Male | 77,048 | 46.1 | 21,627 | 48.6 |
| Female | 89,955 | 53.9 | 22,845 | 51.4 |
|  |  |  |  |  |
| **Age** |  |  |  |  |
| 19–29 | 17,285 | 10.4 | 5,713 | 12.8 |
| 30–39 | 20,582 | 12.3 | 7,078 | 15.9 |
| 40–49 | 27,271 | 16.3 | 9,047 | 20.3 |
| 50–59 | 34,052 | 20.4 | 9,502 | 21.4 |
| 60–69 | 33,110 | 19.8 | 7,247 | 16.3 |
| >70 | 34,703 | 20.8 | 5,885 | 13.2 |
|  |  |  |  |  |
| **Income (KW10,000)** |  |  |  |  |
| Less than 100 | 24,109 | 14.8 | 3,705 | 9.0 |
| 100–199 | 26,428 | 16.3 | 5,462 | 13.3 |
| 200–299 | 25,201 | 15.5 | 6,440 | 15.7 |
| 300–399 | 24,059 | 14.8 | 6,723 | 16.4 |
| 400–499 | 18,707 | 11.5 | 5,220 | 12.7 |
| 500–599 | 19,650 | 12.1 | 6,050 | 14.7 |
| >600 | 24,268 | 14.9 | 7,489 | 18.2 |
|  |  |  |  |  |
| **Education** |  |  |  |  |
| Less than elementary school | 37,496 | 22.5 | 5,633 | 12.7 |
| Less than middle school | 20,239 | 12.1 | 4,228 | 9.5 |
| Less than high school | 49,109 | 29.4 | 14,162 | 32.0 |
| Less than college | 54,460 | 32.7 | 18,389 | 41.5 |
| Graduate school or higher | 5,490 | 3.3 | 1,889 | 4.3 |
|  |  |  |  |  |
| **Occupation** |  |  |  |  |
| Management‧profession | 17,236 | 10.3 | 5,872 | 13.2 |
| Office worker | 14,657 | 8.8 | 5,314 | 12.0 |
| Sales‧service | 20,882 | 12.5 | 6,563 | 14.8 |
| Agriculture‧forestry‧fishing | 19,120 | 11.5 | 3,353 | 7.6 |
| Manipulation of technical‧devices | 15,983 | 9.6 | 4,596 | 10.4 |
| Simple labor | 16,140 | 9.7 | 3,420 | 7.7 |
| Soldier | 574 | 0.3 | 154 | 0.3 |
| Others (housewife, student, or no occupation) | 62,246 | 37.3 | 15,074 | 34.0 |
|  |  |  |  |  |
| **Marital status** |  |  |  |  |
| Married (with spouse) | 114,710 | 68.7 | 30,187 | 68.1 |
| Other (widow, divorce, etc.) | 27,079 | 16.2 | 5,466 | 12.3 |
| Unmarried | 25,062 | 15.0 | 8,671 | 19.6 |
|  |  |  |  |  |
| **Area** |  |  |  |  |
| Dong | 95,776 | 57.3 | 28,877 | 64.9 |
| Eup/Myeon | 71,227 | 42.7 | 15,595 | 35.1 |
|  |  |  |  |  |
| **Generation type** |  |  |  |  |
| 1st generation | 78,933 | 47.3 | 17,967 | 40.4 |
| 2nd generation | 76,396 | 45.7 | 23,029 | 51.8 |
| 3rd generation and over | 11,671 | 7.0 | 3,441 | 7.7 |
|  |  |  |  |  |
| **Self-reported obesity** |  |  |  |  |
| Obesity (25≦BMI) | 51,135 | 30.7 | 14,224 | 32.0 |
| Normal (18.5≦BMI<25) | 108,845 | 65.2 | 28,444 | 64.0 |
| Underweight (BMI<18.5) | 6,852 | 4.1 | 1,786 | 4.0 |

*Participants who provided both self-reported and measured values, including those who had outlier values

**Table 2. Comparison of self-reported and measured height, weight, and body mass index values**

|  | Self-reported | | | Measured | | | Difference* | | |
| --- | --- | --- | --- | --- | --- | --- | --- | --- | --- |
|  | N | Mean | SE | N | Mean | SE | Mean | 95% CI | p-value |
| **Height (cm)** |  |  |  |  |  |  |  |  |  |
| **Male** | 78,260 | 171.6 | 2.9 | 78,260 | 171.0 | 3.1 | −0.59 | −0.61 to −0.57 | <0.0001 |
| 19–29 | 8,515 | 174.5 | 7.0 | 8,515 | 174.5 | 7.2 | −0.03 | −0.07 to 0.00 | 0.0678 |
| 30-39 | 9,866 | 174.8 | 6.3 | 9,866 | 174.5 | 6.5 | −0.26 | −0.30 to −0.22 | <0.0001 |
| 40-49 | 13,016 | 172.8 | 5.8 | 13,016 | 172.3 | 6.0 | −0.43 | −0.47 to −0.39 | <0.0001 |
| 50-59 | 15,459 | 170.3 | 5.5 | 15,459 | 169.7 | 5.7 | −0.65 | −0.68 to −0.62 | <0.0001 |
| 60-69 | 15,420 | 168.2 | 5.8 | 15,420 | 167.1 | 6.1 | −1.02 | −1.06 to −0.98 | <0.0001 |
| ≥70 | 15,984 | 166.3 | 6.3 | 15,984 | 164.6 | 6.5 | −1.70 | −1.77 to −1.64 | <0.0001 |
|  |  |  |  |  |  |  |  |  |  |
| **Female** | 93,124 | 158.6 | 2.6 | 93,124 | 157.9 | 2.8 | −0.71 | −0.73 to −0.69 | <0.0001 |
| 19–29 | 9,437 | 161.5 | 6.2 | 9,437 | 161.6 | 6.3 | 0.11 | 0.08, 0.14 | <0.0001 |
| 30–39 | 11,462 | 161.6 | 5.5 | 11,462 | 161.4 | 5.7 | −0.20 | −0.22 to −0.17 | <0.0001 |
| 40–49 | 15,054 | 160.0 | 5.1 | 15,054 | 159.6 | 5.2 | −0.39 | −0.42 to −0.36 | <0.0001 |
| 50–59 | 19,276 | 157.8 | 4.6 | 19,276 | 157.0 | 4.8 | −0.75 | −0.78 to −0.72 | <0.0001 |
| 60–69 | 18,336 | 155.9 | 4.9 | 18,336 | 154.6 | 5.1 | −1.30 | −1.35 to −1.26 | <0.0001 |
| ≥70 | 19,559 | 153.1 | 6.0 | 19,559 | 150.9 | 5.8 | −2.17 | −2.24 to −2.10 | <0.0001 |
| **Weight (kg)** |  |  |  |  |  |  |  |  |  |
| **Male** | 80,502 | 72.0 | 5.4 | 80,502 | 72.5 | 5.7 | 0.55 | 0.53–0.58 | <0.0001 |
| 19–29 | 8,584 | 73.6 | 16.7 | 8,584 | 74.2 | 17.4 | 0.59 | 0.53–0.65 | <0.0001 |
| 30–39 | 10,025 | 77.1 | 12.9 | 10,025 | 77.8 | 13.4 | 0.72 | 0.67–0.78 | <0.0001 |
| 40–49 | 13,236 | 74.2 | 11.2 | 13,236 | 74.8 | 11.4 | 0.67 | 0.62–0.71 | <0.0001 |
| 50–59 | 15,788 | 70.9 | 9.8 | 15,788 | 71.4 | 10.2 | 0.58 | 0.53–0.62 | <0.0001 |
| 60–69 | 15,833 | 68.0 | 9.5 | 15,833 | 68.5 | 9.8 | 0.51 | 0.45–0.56 | <0.0001 |
| ≥70 | 17,036 | 64.2 | 9.8 | 17,036 | 64.3 | 10.3 | 0.06 | 0.00–0.13 | 0.0687 |
|  |  |  |  |  |  |  |  |  |  |
| **Female** | 97,337 | 57.3 | 3.8 | 97,337 | 58.0 | 4.0 | 0.67 | 0.65–0.69 | <0.0001 |
| 19–29 | 9,106 | 55.9 | 11.8 | 9,106 | 56.6 | 12.8 | 0.67 | 0.61–0.72 | <0.0001 |
| 30–39 | 11,086 | 57.8 | 10.0 | 11,086 | 58.6 | 10.5 | 0.74 | 0.70–0.78 | <0.0001 |
| 40–49 | 14,679 | 57.9 | 8.5 | 14,679 | 58.7 | 9.0 | 0.78 | 0.74–0.82 | <0.0001 |
| 50–59 | 19,377 | 58.2 | 7.2 | 19,377 | 59.0 | 7.6 | 0.75 | 0.72–0.79 | <0.0001 |
| 60–69 | 19,179 | 58.1 | 7.8 | 19,179 | 58.7 | 8.2 | 0.64 | 0.60–0.69 | <0.0001 |
| ≥70 | 23,910 | 55.4 | 7.7 | 23,910 | 55.7 | 8.0 | 0.37 | 0.32–0.42 | <0.0001 |
|  |  |  |  |  |  |  |  |  |  |
| **BMI (kg/m^2^)** |  |  |  |  |  |  |  |  |  |
| **Male** | 76,771 | 24.4 | 1.6 | 76,771 | 24.7 | 1.7 | 0.35 | 0.35–0.36 | <0.0001 |
| 19–29 | 8,321 | 24.1 | 5.1 | 8,321 | 24.3 | 5.3 | 0.20 | 0.18–0.22 | <0.0001 |
| 30–39 | 9,670 | 25.2 | 3.8 | 9,670 | 25.5 | 4.0 | 0.31 | 0.29–0.33 | <0.0001 |
| 40–49 | 12,812 | 24.8 | 3.4 | 12,812 | 25.2 | 3.5 | 0.35 | 0.33–0.37 | <0.0001 |
| 50–59 | 15,217 | 24.4 | 2.9 | 15,217 | 24.8 | 3.1 | 0.38 | 0.37–0.40 | <0.0001 |
| 60–69 | 15,158 | 24.0 | 2.9 | 15,158 | 24.5 | 3.1 | 0.48 | 0.45–0.50 | <0.0001 |
| ≥70 | 15,593 | 23.2 | 3.2 | 15,593 | 23.7 | 3.3 | 0.49 | 0.46–0.52 | <0.0001 |
|  |  |  |  |  |  |  |  |  |  |
| **Female** | 89,649 | 22.8 | 1.5 | 89,649 | 23.3 | 1.6 | 0.49 | 0.48–0.50 | <0.0001 |
| 19–29 | 8,912 | 21.4 | 4.2 | 8,912 | 21.7 | 4.4 | 0.22 | 0.20–0.24 | <0.0001 |
| 30–39 | 10,843 | 22.1 | 3.7 | 10,843 | 22.5 | 3.9 | 0.34 | 0.32–0.36 | <0.0001 |
| 40–49 | 14,370 | 22.6 | 3.2 | 14,370 | 23.1 | 3.4 | 0.42 | 0.40–0.44 | <0.0001 |
| 50–59 | 18,725 | 23.4 | 2.7 | 18,725 | 23.9 | 2.9 | 0.53 | 0.52–0.55 | <0.0001 |
| 60–69 | 17,837 | 23.9 | 3.1 | 17,837 | 24.6 | 3.3 | 0.68 | 0.65–0.70 | <0.0001 |
| ≥70 | 18,962 | 23.8 | 3.2 | 18,962 | 24.6 | 3.4 | 0.83 | 0.80–0.87 | <0.0001 |

*Difference=measured value minus self-reported value

SE: standard Error, CI: confidence interval

**Table 3. Agreement between measured and self-reported BMI values per category**

| **Measured**  **BMI** | **Self-reported BMI** | | | | | **Agreement** | **Kappa**  **(p-value)** |
| --- | --- | --- | --- | --- | --- | --- | --- |
|  | **Underweight** | **Normal** | **Overweight** | **Obesity** | **Total** |  |  |
| **Total** | 6,836 (100.0) | 67,439 (100.0) | 41,186 (100.0) | 50,959 (100.0) | 166,420 |  |  |
| Underweight | 5,047 (73.8) | 1,765 (2.6) | 37(0.1) | 17 (0.0) | 6,866 | 79.6% | 0.82  (<0.0001) |
| Normal | 1,703 (24.9) | 55,158(81.8) | 3,991(9.7) | 493 (1.0) | 61,345 |  |  |
| Overweight | 51 (0.7) | 9,210 (13.7) | 27,736(67.3) | 3,280 (6.4) | 40,277 |  |  |
| Obesity | 35 (0.5) | 1,306 (1.9) | 9,422(22.9) | 47,169(92.6) | 57,932 |  |  |
| **Male** | 1,983 (100.0) | 25,600 (100.0) | 21,315(100.0) | 27,873 (100.0) | 76,771 |  |  |
| Underweight | 1,403 (70.8) | 557 (2.2) | 20(0.1) | 7 (0.0) | 1,987 | 80.5% | 0.82  (<0.0001) |
| Normal | 554 (27.9) | 19,665 (76.8) | 1,411(6.6) | 196 (0.7) | 21,826 |  |  |
| Overweight | 14 (0.7) | 4,880 (19.1) | 14,500(68.0) | 1,438(5.2) | 20,832 |  |  |
| Obesity | 12 (0.6) | 498 (1.9) | 5,384(25.3) | 26,232(94.1) | 32,126 |  |  |
| **Female** | 4,853 (100.0) | 41,839 (100.0) | 19,871(100.0) | 23,086(100.0) | 89,649 |  |  |
| Underweight | 3,229 (66.5) | 657(1.6) | 11(0.1) | 8(0.0) | 3,905 | 78.8% | 0.80  (<0.0001) |
| Normal | 1,549 (31.9) | 33,437 (79.9) | 1,333 (6.7) | 232 (1.0) | 36,551 |  |  |
| Overweight | 48 (1.0) | 6,643 (15.9) | 12,184(61.3) | 1,069 (4.6) | 19,944 |  |  |
| Obesity | 27 (0.6) | 1,102 (2.6) | 6,343 (31.9) | 21,777 (94.3) | 29,249 |  |  |

BMI: body mass index

Underweight (<18.5), normal (18.5–22.9), overweight (23.0–24.9), and obesity (≥25.0)

**Figure 1. Correlation distribution and Bland-Altman plot of the measured and self-reported height values**


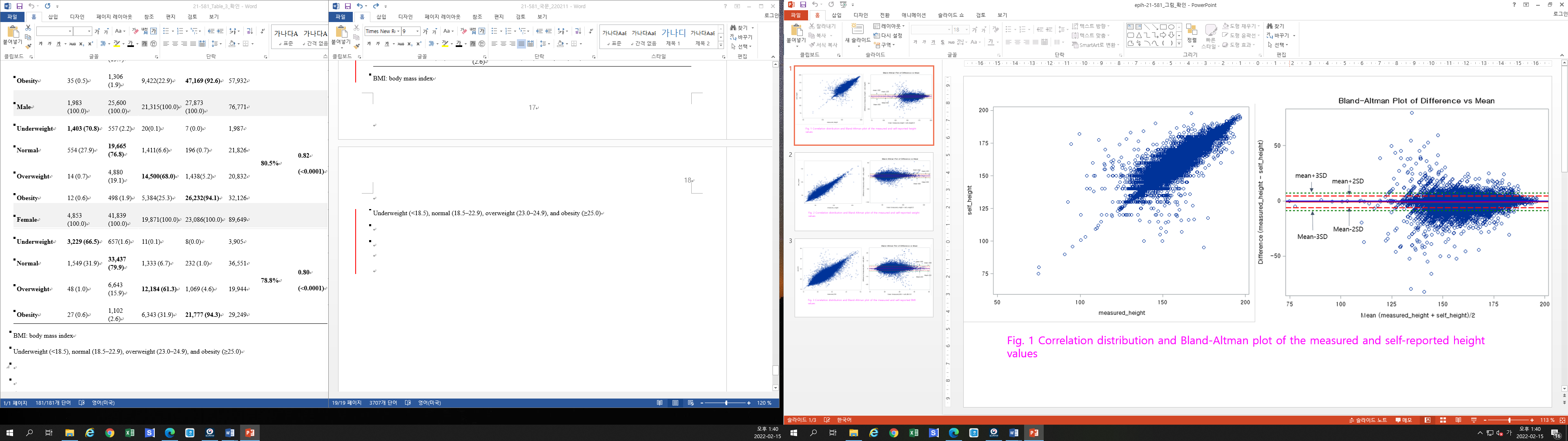


**Figure 2. Correlation distribution and Bland-Altman plot of the measured and self-reported weight values**


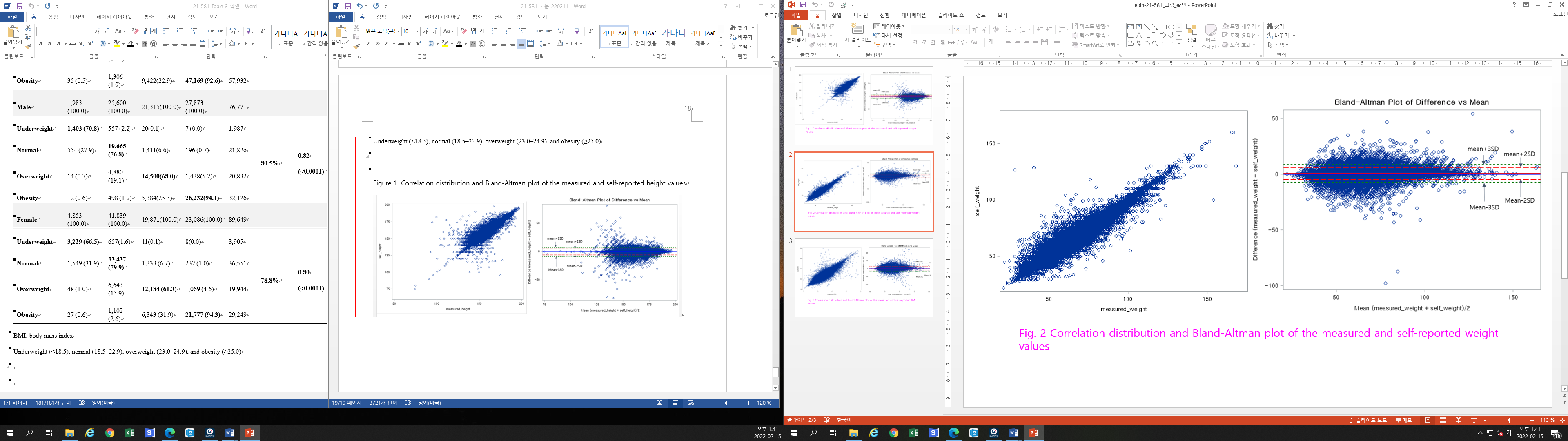


**Figure 3. Correlation distribution and Bland-Altman plot of the measured and self-reported BMI values**


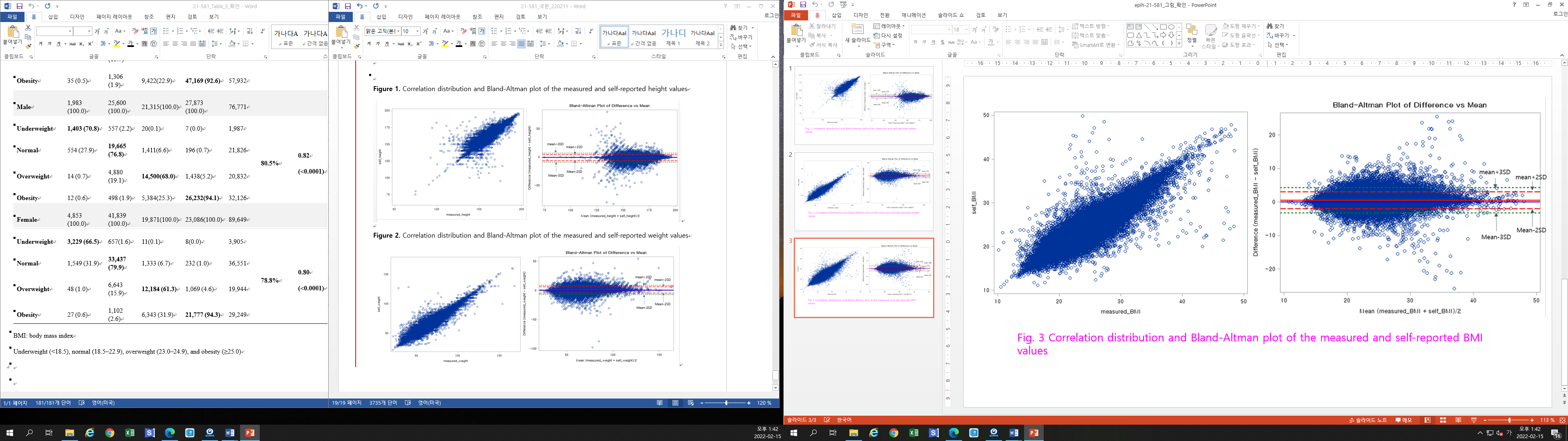

Supplement: Supplementary file 1 [file epih-44-e2022024-suppl.docx]
